# Supplementary material for: Tunable and scalable fabrication of block copolymer-based 3D polymorphic artificial cell membrane array
Source: Nat Commun. 2022 Mar 10;13:1261. doi: 10.1038/s41467-022-28960-y (PMC8913694; doi:10.1038/s41467-022-28960-y)
Supplement: Supplementary file 1 — Supplementary Information [file 41467_2022_28960_MOESM1_ESM.pdf]

[Supplementary information]

Tunable and scalable fabrication of block copolymer-based 3D polymorphic artificial cell membrane array

*Dong-Hyun Kang, Won Bae Han, Hyun Il Ryu, Nam Hyuk Kim, Tae Young Kim, Nakwon Choi, Ji Yoon Kang, Yeon Gyu Yu, and Tae Song Kim**

Theory

To explain the morphology dynamics of 3DBCPMs according to the AC frequency and amplitude of electric field, we adopted the theory of electro-deformation of a spheroidal vesicle in an AC electric field. Although the 3DBCPM is immobilized and rooted in the Si microwell, it has a unilamellar membrane and can be considered to be similar to a spheroidal vesicle in the adopted model. The earliest theoretical models of vesicle electro-deformation were based on minimizing the total surface energy, consisting of the mechanical energy (from tension and bending) and electrical energy (from Maxwell stresses) of the membrane. Further details can be found in previous studies[1-3].

When an electric field is applied to a thin membrane, it generates electrohydrodynamic flow with the same symmetry as the induced electric stresses. The corresponding fluid velocity, which is responsible for a closed unilamellar membrane (quasi-spherical shape), is given by

$$C^{\mathrm{el}} = 8\sqrt{\frac{\pi}{5}}\frac{6p^{\mathrm{el}}-\tau^{\mathrm{el}}}{23\chi+32}. \qquad (1)$$

The radial and tangential electric stresses are given by

$$p^{\mathrm{el}} = \frac{1}{32\pi}[-2(\tau_1^2+\tau_2^2)S + 5\tau_3^2 - 2d^{\infty}\tau_3 + 5\tau_4^2 + 2(d^{\infty})^2], \qquad (2)$$

$$\tau^{\mathrm{el}} = \frac{1}{8\pi}[(\tau_1^2+\tau_2^2)S + 2\tau_3^2 + (d^{\infty})\tau_3 + 2\tau_4^2 - (d^{\infty})^2], \qquad (3)$$

where $\tau_1 = Re[P^{\mathrm{in}}]$, $\tau_2 = Im[P^{\mathrm{in}}]$, $\tau_3 = Re[P^{\mathrm{ex}}]$, and $\tau_4 = Im[P^{\mathrm{ex}}]$. Further, *Re*[ ] and *Im*[ ] denote the real and imaginary parts, respectively.

$$P^{\mathrm{ex}} = d^{\infty}\frac{(-k_{\mathrm{in}}+k_{\mathrm{ex}})+k_{\mathrm{in}}V_{\mathrm{m}}}{k_{\mathrm{in}}+2k_{\mathrm{ex}}},\ P^{\mathrm{in}} = d^{\infty}k_{\mathrm{ex}}\frac{3-2V_{\mathrm{m}}}{k_{\mathrm{in}}+2k_{\mathrm{ex}}}, \qquad (4)$$

where k_{in} and k_{ex} denote the dimensionless complex conductivities of the interior and exterior fluids, respectively.

$$k_{\mathrm{in}} = \Lambda + \mathrm{i}\omega\mathrm{S},\ k_{\mathrm{ex}} = 1 + \mathrm{i}\omega, \qquad (5)$$

where the ratios of the electric properties of the interior and exterior fluids are given by

$$\Lambda = \frac{\lambda_{\mathrm{in}}}{\lambda_{\mathrm{ex}}},\ \mathrm{S} = \frac{\varepsilon_{\mathrm{in}}}{\varepsilon_{\mathrm{ex}}} \qquad (6)$$

where λ_{in} and λ_{ex} and $\varepsilon_{\mathrm{in}}$ and $\varepsilon_{\mathrm{ex}}$ denote the conductivities and permittivities of the interior and exterior fluids, respectively.

The transmembrane potential V_m is given by

$$V_{\mathrm{m}}(\omega) = \frac{3}{2}\frac{1}{1+(G_{\mathrm{m}}+\mathrm{i}\omega C_{\mathrm{m}})(\frac{1}{\Lambda}+\frac{1}{2})}, \quad (7)$$

where G_m and C_m are the dimensionless membrane conductivity and capacitance per unit area, respectively.

The viscosity parameter χ is defined as

$$\chi = \frac{\eta_{\mathrm{in}}}{\eta_{\mathrm{ex}}}, \quad (8)$$

where η_{in} and η_{ex} are the internal and external viscosities, respectively.

Because electric stresses directly affect only the ellipsoidal j=2, m=0 mode, the most important contribution to the structural deformation comes from the elongational f_{20} mode.

$$f_{20}^{\max} = \sqrt{\frac{\Delta}{2}}. \quad (9)$$

The access area is redistributed among all shape modes as

$$\Delta = \frac{\mathrm{A}}{a^2} - 4\pi = \sum_{\mathrm{j}=2}^{\infty}\sum_{\mathrm{m}=-\mathrm{j}}^{\mathrm{j}}\frac{(-1)^m}{2}(j-1)(j+2)f_{\mathrm{jm}}f_{\mathrm{j-m}}. \quad (10)$$

The shape evolution strongly depends on the effective tension, σ_h. For a quasi-spherical vesicle, using the relation between the excess area and the shape modes (Eq. 10), and including only the dominant contribution from the f_{20} mode, the effective tension σ_h can be written as

$$\sigma_{\mathrm{h}} = \sigma_0 \exp\left(\frac{2\kappa\bar{\Delta}}{k_{\mathrm{B}}T}\right) = \sigma_0 \exp\left(\frac{4\kappa}{k_{\mathrm{B}}T}f_{20}^2\right), \quad (11)$$

where σ_0 is the initial tension in the membrane, κ is the bending rigidity, k_B is the Boltzmann constant, and T is the temperature.

The shape evolution of a vesicle in an AC electric field is described by the following nonlinear equation:

$$\frac{\partial f_{20}}{\partial \mathrm{t}} = C^{\mathrm{el}} - Ca^{-1}\frac{24\left[6+exp\left(\frac{4\kappa}{k_{\mathrm{B}}T}f_{20}^2(t)\right)\right]}{23\chi+32}f_{20}(t), \quad (12)$$

where Ca is the capillary number.

In the steady state, $\frac{\partial f_{20}}{\partial t} = 0$, and the stationary vesicle shape is given by

$$f_{20} = \sqrt{\frac{\pi}{5}}\frac{6p^{\mathrm{el}}-\tau^{\mathrm{el}}}{3(6+\sigma_{\mathrm{h}})}. \quad (13)$$

We calculated the vesicle half-length along the field direction $\frac{\mathrm{a}}{\mathrm{b}} = \frac{a_{max}}{a} = 1 + \sqrt{5/4\pi} f_{20}$ from Eq. 13, using the initial membrane tension σ_0 as the only fitting parameter.

In this study, 3DBCPMs were formed in a 10 mM sucrose solution. Therefore, the conductivities and dielectric constants of the inside and outside of the 3DBCPM were approximately equal (λ_{in} and λ_{ex} = 0.01 mS/m, and $\varepsilon_{\mathrm{in}}$ and $\varepsilon_{\mathrm{ex}}$ = 80). The dimensionless membrane conductivity G_{m} and capacitance C_{m} per unit area were 0.001 S/m^2 and 0.00256 F/m^2, respectively[4]. The calculated values of the aspect ratio of the 3DBCPM are shown in Figure 3b. The reason for the difference between the theoretically estimated and experimentally measured values (aspect ratio) is that, unlike the theoretical model limited to closed vesicles without an additional supply of block copolymer, the 3DBCPM can grow into a high aspect ratio structure by electric field-induced tangential and normal forces until block copolymers sufficiently deposited in microwell are depleted, as shown in the figure below:

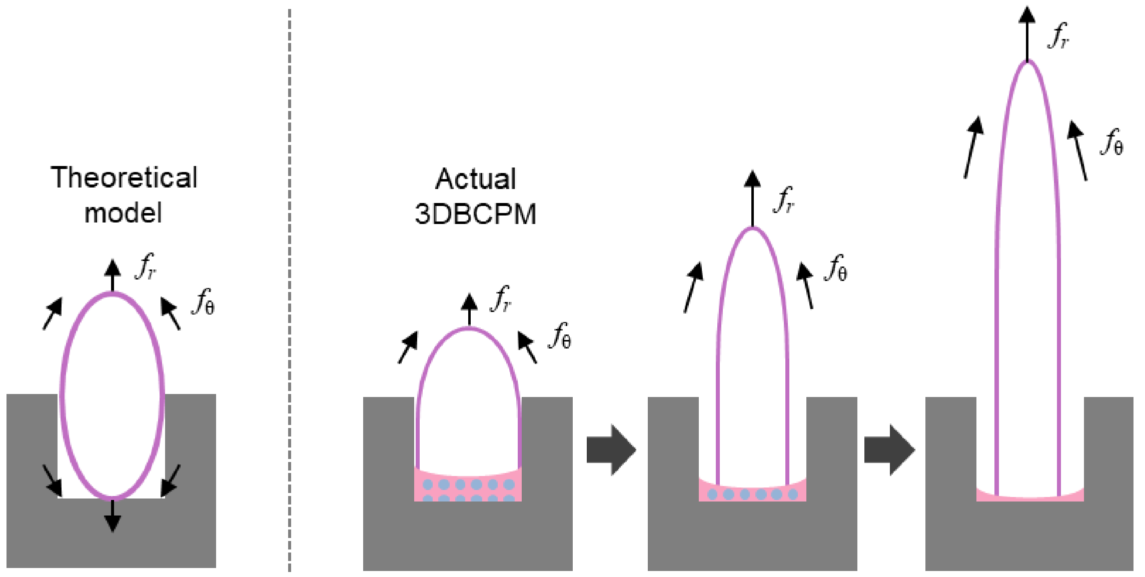

<Growth mechanisms for theoretical model and actual 3DBCPM, where f_r and f_θ represents the electric field-induced normal and tangential forces>

Supplementary figures

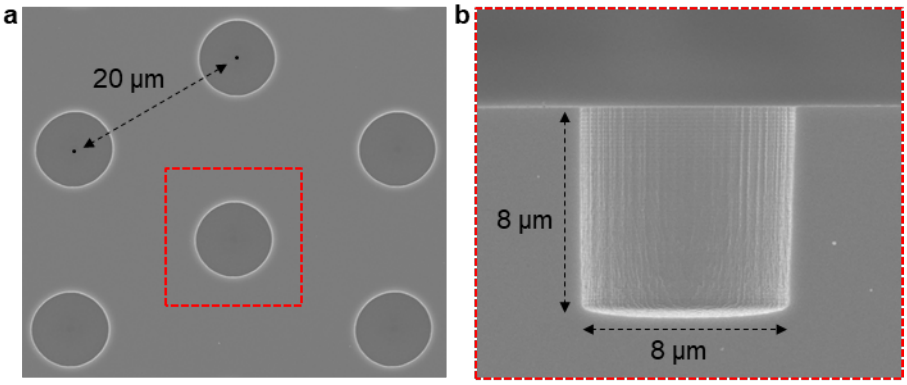

Figure S1. SEM images of a microwell array used in this study.

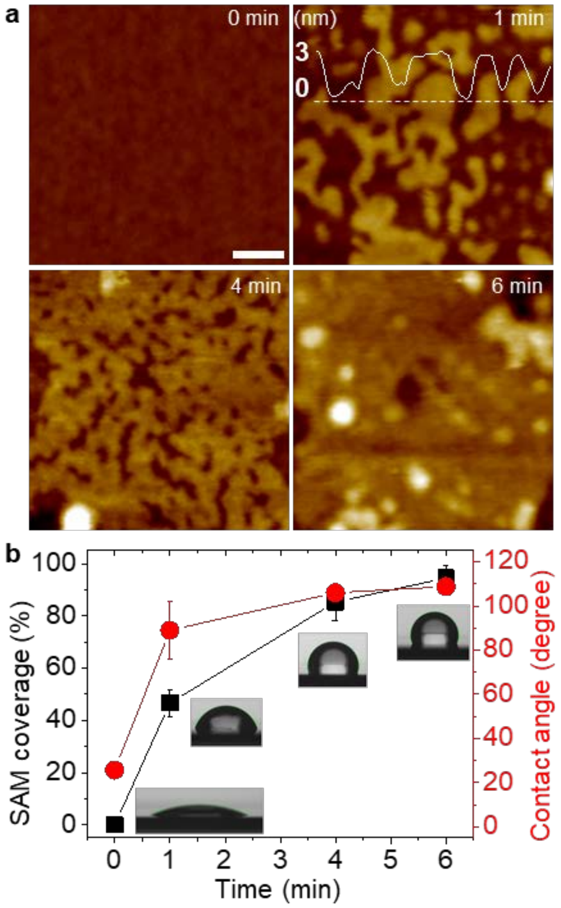

Figure S2. (a) Atomic force microscopy (AFM) images of silicon (Si) templates treated with perfluorododecyltrichlorosilane (PFDDTS) for different durations (0, 1, 4, and 6 min) and the line profile measurement confirming that a monolayer of silane (~3 nm thick) was self-assembled on the substrate. Scale bar: 100 nm. (b) Stamping time-dependent surface coverages of PFDDTS self-assembled monolayer (SAM) and the corresponding water contact angle measurements. Data are presented as means ± standard deviation (n=5 independent samples).

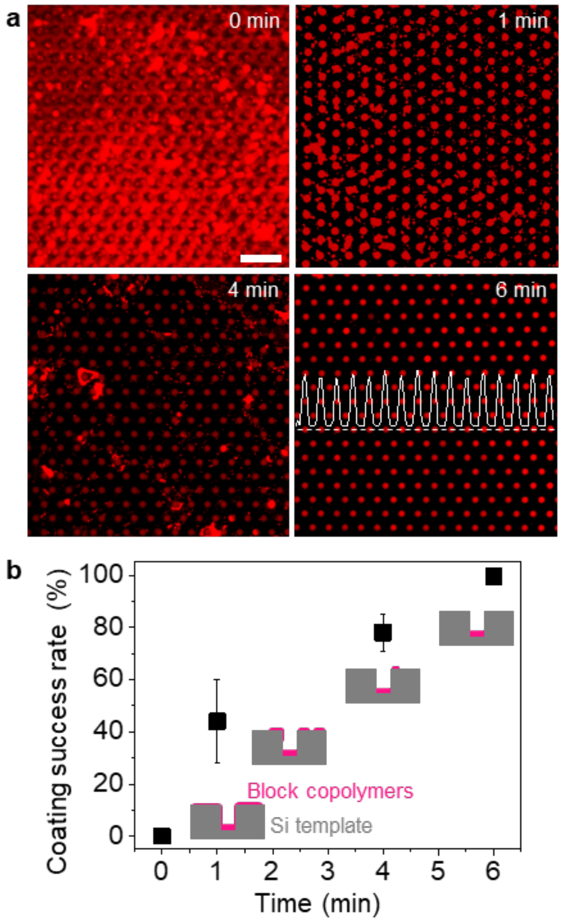

Figure S3. (a) Fluorescence microscopy images of 2.5 wt% of polybutadiene-*b*-polyethyleneoxide (PBd-PEO) coated on Si microwell templates treated with PFDDTS for different durations (0, 1, 4, and 6 min) and the line profile measurement confirming uniform and selective coating of block copolymers onto the microwells. Scale bar: 50 μm. (b) Stamping time-dependent coating success rates defined by the ratio of microwells uniformly coated with block copolymers without residues. Data are presented as means ± standard deviation (n=5 independent samples).

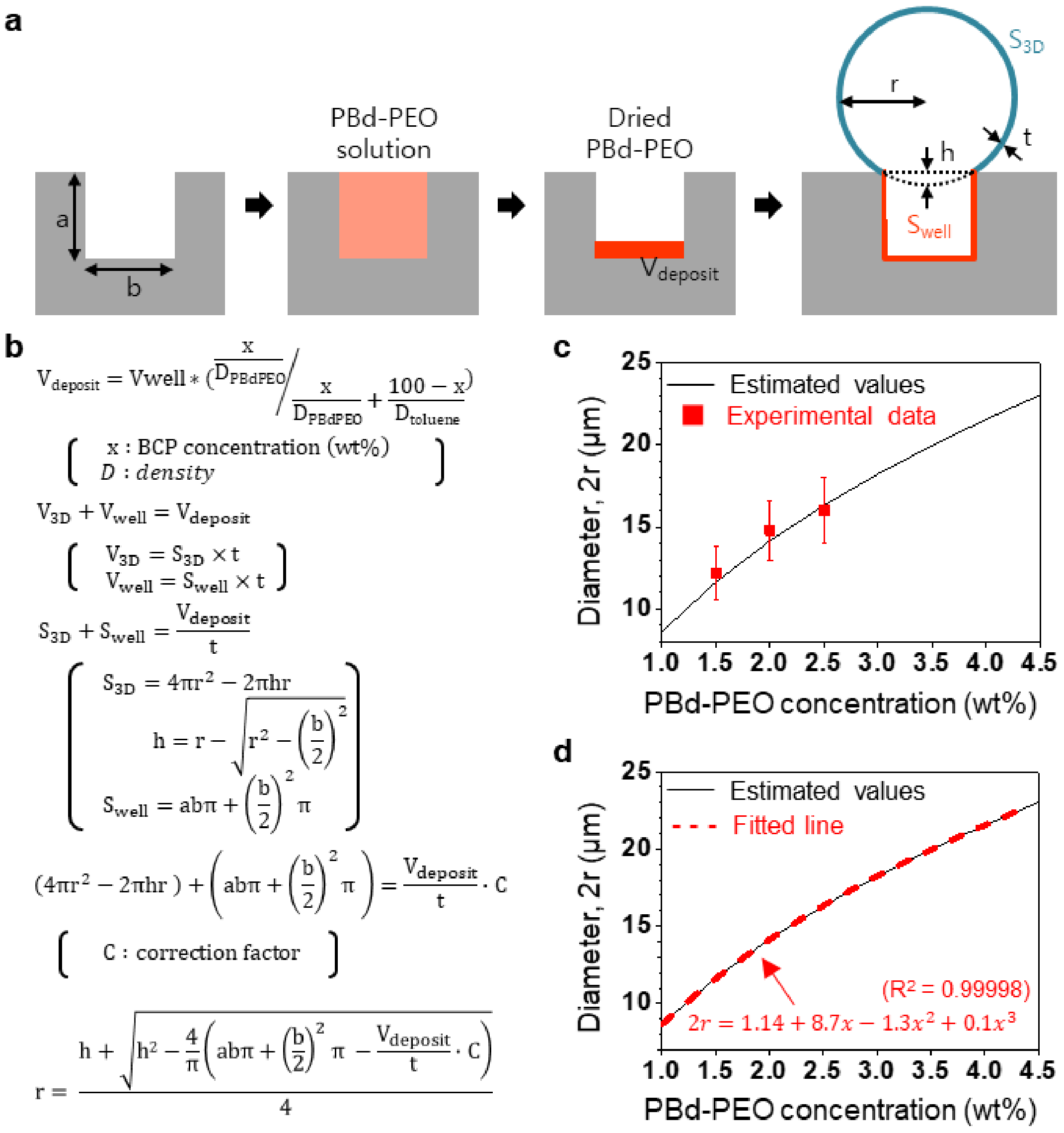

Figure S4. (a) Schematic illustration of the fabrication of spherical 3DBCPM. (b) Estimation of the diameter of spherical 3DBCPMs. (c) Estimated values and experimental data for the diameter of spherical 3DBCPMs as a function of PBd-PEO concentration, where the correction factor of 1.2 was used. Data are presented as means ± standard deviation (n=3 independent samples). (d) Fitting of the estimated values to obtain an equation for determining the diameter of spherical 3DBCPMs.

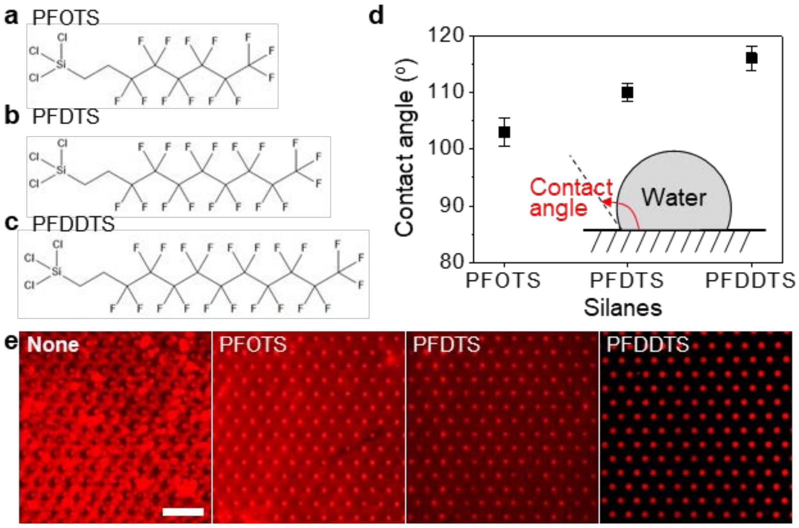

Figure S5. Chemical structures of (a) perfluorooctyltrichlorosilane (PFOTS), (b) perfluorodecyltrichlorosilane (PFDTS), and (c) perfluorododecyltrichlorosilane (PFDDTS). (d) Contact angle measurements of Si templates treated with these three silanes. Data are presented as means ± standard deviation (n=5 independent samples). (e) Fluorescence microscopy images of 2.5 wt% of PBd-PEO coated on Si microwell templates treated with various silanes. Scale bar: 50 μm.

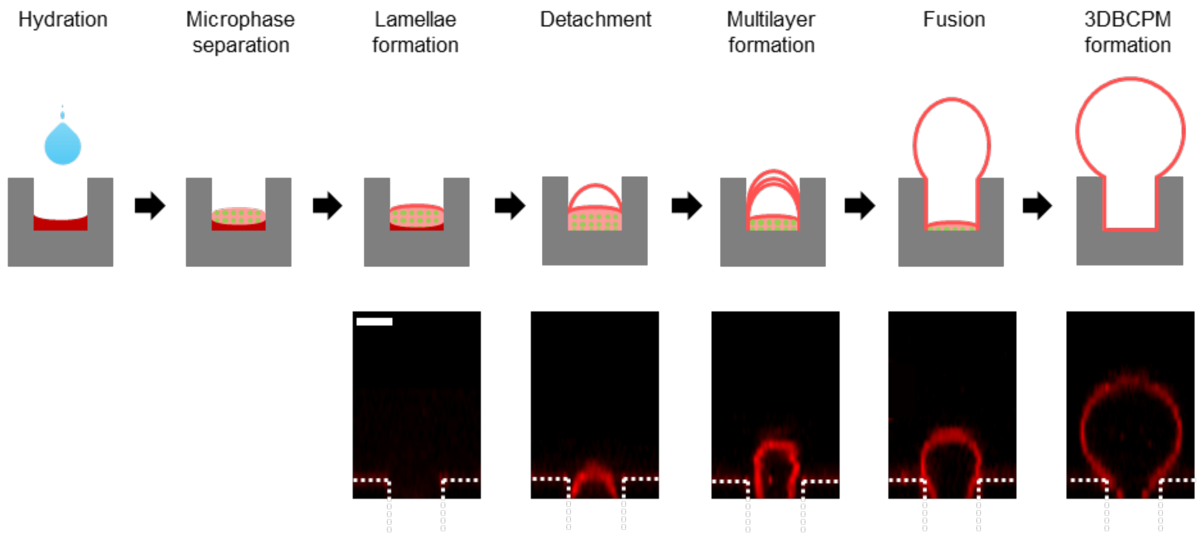

Figure S6. Growth mechanism of 3DBCPMs upon hydration with an electric field. Scale bar: 5 µm.

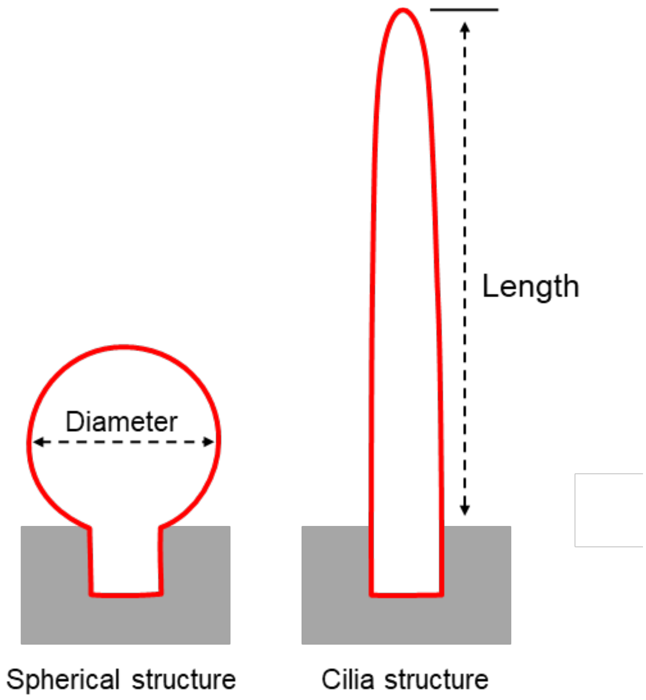

Figure S7. A parameter description for the size of 3DBCPMs.

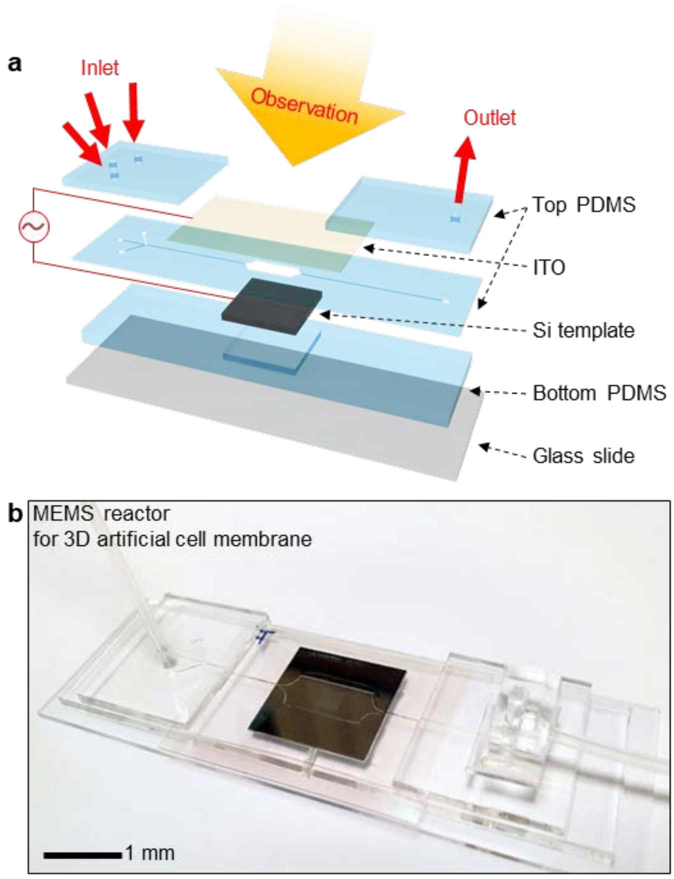

Figure S8. (a) Schematic illustration and (b) optical image of microelectromechanical system (MEMS) reactor for the formation 3DBCPMs.

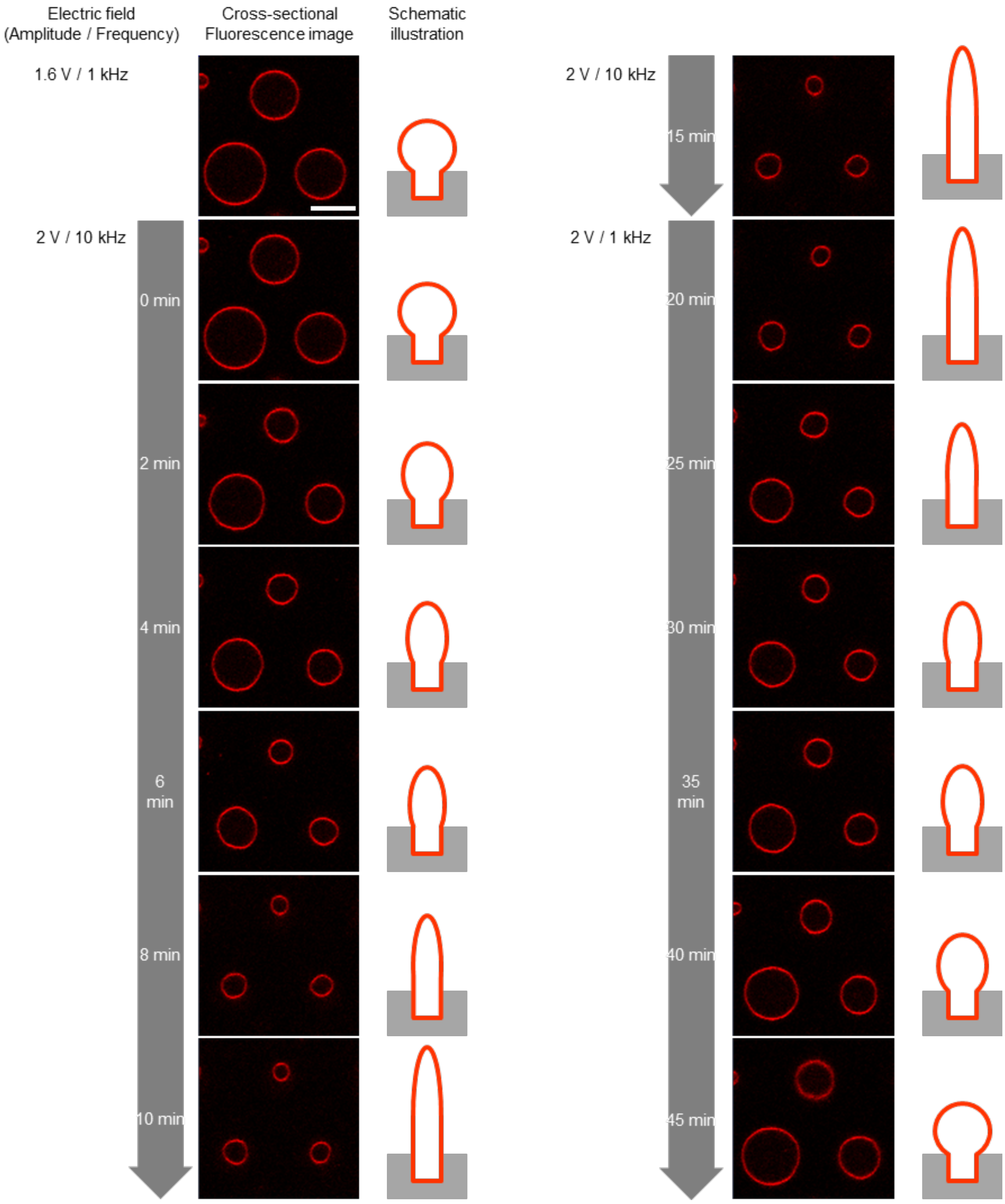

Figure S9. Reversible shape transformation of 3DBCPMs produced with 2 wt% of PBd-PEO by controlling an electric field. Scale bar: 10 μm.

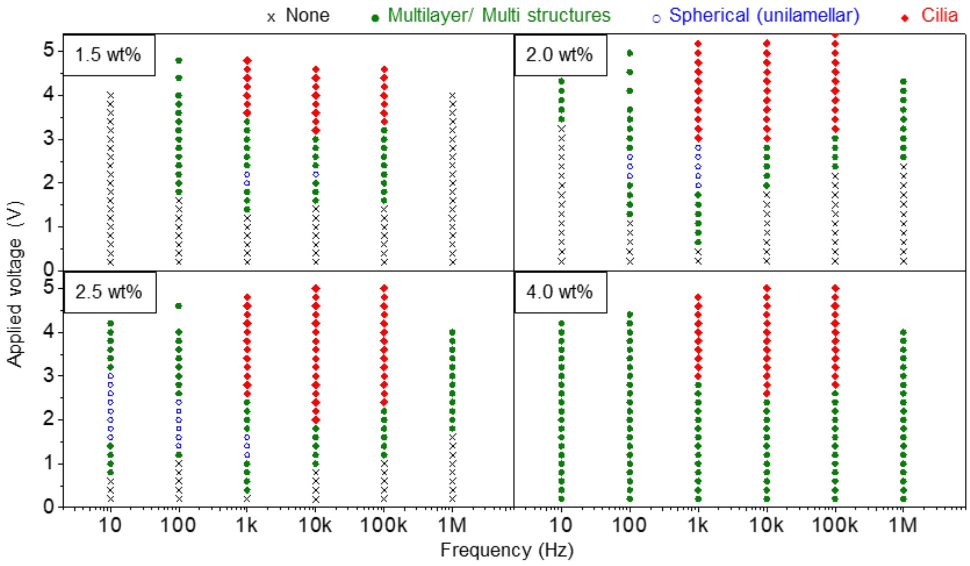

Figure S10. Relationship between 3DBCPM shapes and three factors: block copolymer concentration and frequency and amplitude of electric field.

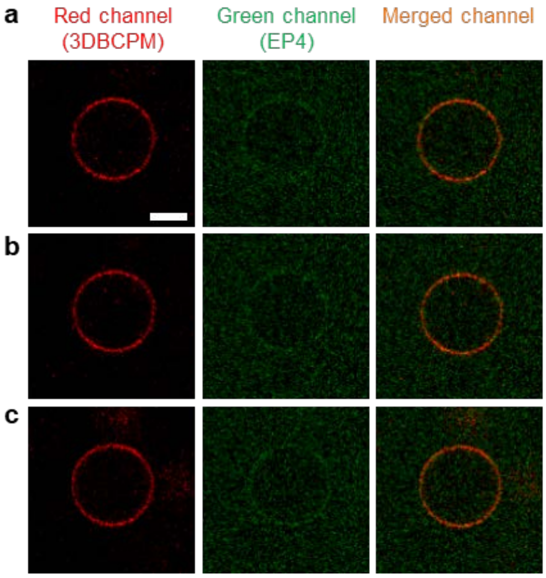

Figure S11. Alexa fluor 488-tagged human prostaglandin E2 receptor 4 (EP4) incorporated on 3DBCPM after (a) thorough washing with 5 mM HEPES buffer (pH 7.4) for 30 min, (b) acidic pH shock with the HEPES buffer (pH 3.1), and (c) basic pH shock with the HEPES buffer (pH 11.8). The unchanged green fluorescence intensity on the membrane of 3DBCPM indicates proper integration of the proteins onto 3DBCPM. Scale bar: 5 μm

Mechaelis-Menten Equation

For the application of 3DBCPMs, we mimicked human intestinal organs by incorporating β-Ga. The introduced FDG was converted into two galactose and a GFP by reaction with the β-Ga. This enzymatic reaction was evaluated by the enzyme kinetics.

An enzyme, E, promotes a reaction of the substrate, S, to produce a product, P. The enzymatic chemical reaction can be expressed as:

$$\mathrm{E} + \mathrm{S} \rightleftharpoons \mathrm{ES} \xrightarrow{k_{\mathrm{cat}}} \mathrm{E} + \mathrm{P} \qquad (1)$$

, where k_{cat} is the catalytic rate constant.

According to the Michaelis-Menten Equation, the reaction rate v can be described as:

$$v = \frac{\mathrm{d[P]}}{\mathrm{d}t} = V_{\mathrm{max}} \frac{[\mathrm{S}]}{K_{\mathrm{M}} + [\mathrm{S}]} = k_{\mathrm{cat}}[\mathrm{E}]_0 \frac{[\mathrm{S}]}{K_{\mathrm{M}} + [\mathrm{S}]} \qquad (2)$$

, where [P], [S], and $[E]_0$ are the concentrations of product, substrate, and enzyme, respectively, V_{max} is the maximum rate, and K_M is the Michaelis constant.

Since the [S] was very high as 125 μM in our experiments, the reaction rate in the initial rate period was approximately equal to the V_{max}. Therefore, Equation (2) can be simplified as

$$v_{\mathrm{initial}} \approx V_{\mathrm{max}} = k_{\mathrm{cat}}[\mathrm{E}]_0 \qquad (3)$$

Since β-Ga bound to EDC conjugated to 1% DSPE-PEG-NHS in 3DBCPMs, the [E] was proportional to the surface area of 3DBCPMs. Because k_{cat} was a constant in the same kind of enzymatic reaction, V_{max} depended on $[E]_0$. Thus, we can calculate proportional V_{max} followed different types of 3DBCPMs using surface area of them as shown in Fig. S12 (red hollow circle). And, the [P] was associated with the fluorescence intensity as shown in Fig. 5e, so the slope of the initial rate period in Fig. 5e could be expressed as $v_{initial}$, which is derived by experimental results (Fig. S12). The values for spherical and cilia structures were normalized by those of planar structure.

According to Equation (3), $v_{initial}$ should be close to V_{max}, but $v_{initial}$ of 3DBCPMs obtained from the experimental results was higher than V_{max} derived by calculations. Therefore, we supposed that this behavior was originated from the 3D architecture of the spherical and cilia structures proposed in this work that was favorable for active and effective reactions and from the aforementioned large surface area.

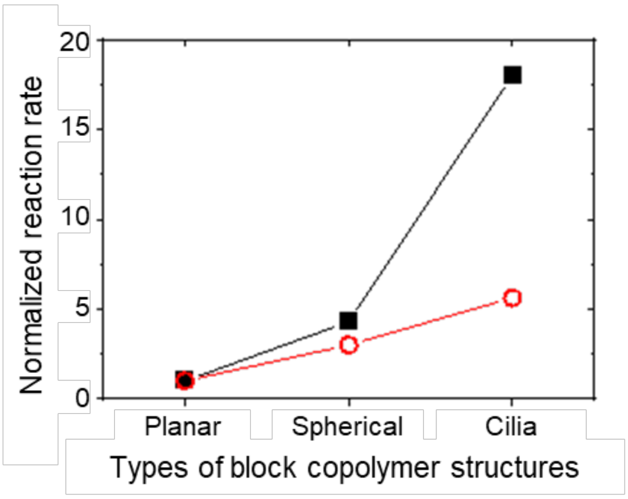

Figure S12. Enzymatic reaction rates of three different block copolymer structures. Black squares (■) are $v_{initial}$ obtained from the experimental results in Fig. 5e, and red hollow circles (○) are V_{max} derived by calculations

References

1. Vlahovska, P. M., Gracià, R. S., Aranda-Espinoza, S. & Dimova, R. Electrohydrodynamic Model of Vesicle Deformation in Alternating Electric Fields. *Biophys. J.* **96**, 4789–4803 (2009).

2. Nganguia, H. & Young, Y.-N. Equilibrium electrodeformation of a spheroidal vesicle in an ac electric field. *Phys. Rev. E* **88**, 052718 (2013).

3. Dimova, R., Bezlyepkina, N., Jordo, M. D., Knorr, R. L., Riske, K. A., Staykova, M., Vlahovska, P. M., Yamamoto, T., Yang, P. & Lipowsky, R. Vesicles in electric fields: Some novel aspects of membrane behavior. *Soft Matter* **5**, 3201-3212 (2009).

4. Salipante, P. F., Knorr, R. L., Dimova, R. & Vlahovska, P. M. Electrodeformation method for measuring the capacitance of bilayer membranes. *Soft Matter* **8**, 3810-3816 (2012).
